# Development of Intraoperative Near-Infrared Fluorescence Imaging System Using a Dual-CMOS Single Camera

**DOI:** 10.3390/s22155597

**Published:** 2022-07-26

**Authors:** Janghoon Choi, Jun Geun Shin, Hyuk-Sang Kwon, Yoon-Oh Tak, Hyeong Ju Park, Jin-Chul Ahn, Joo Beom Eom, Youngseok Seo, Jin Woo Park, Yongdoo Choi, Jonghyun Eom

**Affiliations:** 1 Intelligent Photonic IoT Research Center, Korea Photonics Technology Institute, Gwangju 61007, Korea; janguri@kopti.re.kr (J.C.); yoonohtak@kopti.re.kr (Y.-O.T.); 2 Department of Biomedical Science & Engineering, Gwangju Institute of Science and Technology, Gwangju 61005, Korea; hyuksang@gist.ac.kr; 3 Optical Precision Measurement Research Center, Korea Photonics Technology Institute, Gwangju 61007, Korea; jgshin354@kopti.re.kr; 4 Medical Laser Research Center, Dankook University, Cheonan 31116, Korea; piotex@hanmail.net (H.J.P.); jcahn@dankook.ac.kr (J.-C.A.); 5 College of Medicine, Dankook University, Cheonan 31116, Korea; jbeom@dankook.ac.kr; 6 WONTECH Co., Ltd., Daejeon 34028, Korea; physys@wtlaser.com; 7 BioActs Co., Ltd., Incheon 21666, Korea; park@bioacts.com; 8 Research Institute, National Cancer Center, Goyang 10408, Korea; ydchoi@ncc.re.kr

**Keywords:** near-infrared fluorescence imaging system, image-guided surgery, dual-CMOS single camera, lymph node mapping

## Abstract

We developed a single-camera-based near-infrared (NIR) fluorescence imaging device using indocyanine green (ICG) NIR fluorescence contrast agents for image-induced surgery. In general, a fluorescent imaging system that simultaneously provides color and NIR images uses two cameras, which is disadvantageous because it increases the imaging head of the system. Recently, a single-camera-based NIR optical imaging device with quantum efficiency partially extended to the NIR region was developed to overcome this drawback. The system used RGB_NIR filters for camera sensors to provide color and NIR images simultaneously; however, the sensitivity and resolution of the infrared images are reduced by 1/4, and the exposure time and gain cannot be set individually when acquiring color and NIR images. Thus, to overcome these shortcomings, this study developed a compact fluorescent imaging system that uses a single camera with two complementary metal–oxide semiconductor (CMOS) image sensors. Sensitivity and signal-to-background ratio were measured according to the concentrations of ICG solution, exposure time, and camera gain to evaluate the performance of the imaging system. Consequently, the clinical applicability of the system was confirmed through the toxicity analysis of the light source and in vivo testing.

## 1. Introduction

With the development of imaging technology using various optical characteristics of samples, obtaining image information not obtained through visually recognizable visible images is possible [1,2,3,4]. Among these technologies, image acquisition based on near-infrared (NIR) fluorescent images is crucial to the field of life science and medical diagnosis because of its simplicity and robustness. Recently, with the acceleration of the image acquisition system, real-time fluorescent images can be provided, enabling image induction surgery based on intraoperative lesion detection beyond conventional simple fluorescent image-based pathology and imaging diagnosis [5,6].

Intra-operative image-guided surgery based on NIR fluorescent contrast agents (indocyanine green and methylene blue) licensed by the Food and Drug Administration can detect lesions that cannot be visually identified. This technology provides high sensitivity and real-time images and intuitive information through the matching of visible and fluorescent images, and is attracting attention as an imaging device suitable for image-guided surgery [7].

Owing to these advantages, NIR fluorescence imaging technology is used for sentinel lymph node mapping in patients with breast cancer [8,9,10], vulvar cancer [11], cutaneous melanoma [12], bladder cancer [13], parathyroidectomy [14], intraoperative tumor detection [15,16], laparoscopic cholecystectomy [17], and perforator flap reconstruction [18]. Moreover, several NIR fluorescence imaging systems have received FDA 510(k) Class 2 medical device clearance [7]: the SPY imaging system (Novadaq Technologies, Michigan, United States), PDE photodynamic eye (Hamamatsu Photonics K.K., Hamamatsu, Japan), and Fluobeam 800 (Fluoptics, Grenoble, France).

The SPY and Flare imaging systems use two cameras for color and NIR images to provide NIR and color images [5]. However, in such a system, the volume of the image acquisition part is large, which may be inconvenient to the vision or movement of the operator and surgical assistant. Consequently, for compact image acquisition, Chen et al. developed a fluorescent imaging system using a camera with RGB_NIR pattern filters [19]. In a normal color camera, a Bayer-pattern filter is inserted in front of the sensor for color images, and the RGB_NIR pattern filter camera acquires images in red, blue, green, and NIR in four pixels simultaneously. Consequently, the system configuration can be simplified and the image acquisition unit can be manufactured compactly. However, because only one out of four pixels is used for NIR image acquisition, this camera has reduced sensitivity and resolution compared with general cameras. In addition, obtaining optimal color and NIR images simultaneously is challenging because the exposure time and gain of the color and infrared images cannot be adjusted separately.

Thus, to complement the above two camera configurations, this study developed a dual-sensor camera imaging system wherein two complementary metal–oxide semiconductor (CMOS) image sensors were combined with a dichroic prism in one camera module to obtain a color image and an NIR image separately. Further, to evaluate and optimize the performance of the developed system, the optical characteristics of the system, sensitivity and signal-to-background ratio of the fluorescence images according to the indocyanine green (ICG) concentration, and toxicity of the light source were evaluated. In addition, a preclinical test to obtain NIR fluorescence images using mice was conducted to confirm its clinical applicability.

## 2. Materials and Methods

### 2.1. System Design and Development

**Figure 1:**
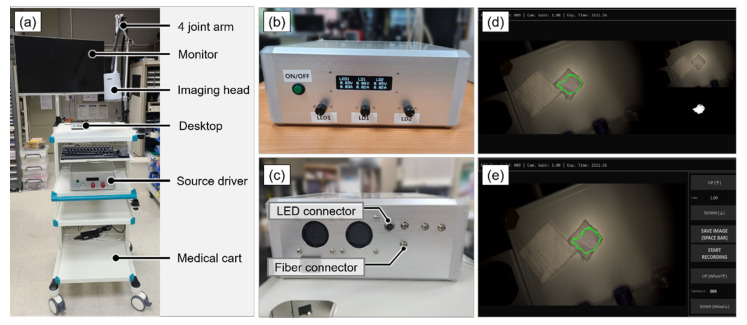
(**a**) NIR fluorescence imaging system. (**b**) Front panel of the source driver. (**c**) Rear panel of the driver. (**d**,**e**) Imaging acquisition program.

Figure 1a shows the overall appearance of the developed NIR fluorescence imaging system and the image acquisition program. The developed imaging system mainly comprised an imaging head, a display monitor, and a medical cart. The medical cart comprised a 4-joint arm that fixes the imaging head, an image-processing desktop for image acquisition and analysis, driving software, and a light-source driver. Figure 1b,c are the light source driver that supplied and controlled the voltage and current to the visible light source white LED and laser diodes for fluorescent excitation. Figure 1d,e show the driving software that was used to control the exposure time, frame rate, contrast, and threshold of the system. In addition, overlay images of visible and NIR fluorescent images and boundary and histogram information of fluorescent signals are provided.

**Figure 2:**
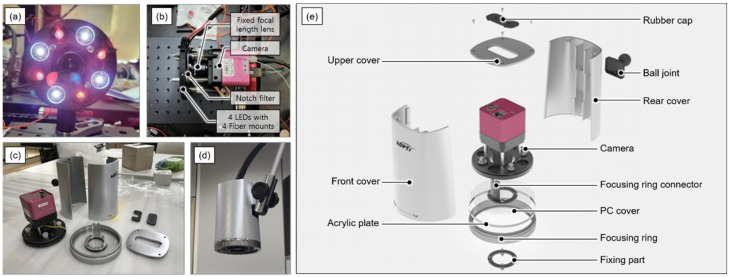
Imaging head of the system. (**a**) illumination plate for 4 white LEDs and 4 fiber mounts (**b**) Illumination and image acquisition parts. (**c**) Parts of imaging head (**d**) Imaging head assembled with four joint arms. (**e**) detailed parts of the imaging head case.

Figure 2 shows a configuration photograph of an image head used to obtain an NIR fluorescent image. The imaging head included an illumination unit and image-acquisition unit. In the lighting unit, a lighting mount with eight ports was configured to illuminate the white LED in the visible light band and NIR fluorescence excitation light source. Further, the visible light source was composed of four white LEDs (W42180, Seoul Semiconductor, Ansan, Korea) and irradiated the sample through four ports on the lighting mount. Two 785 nm laser diodes (WSLX-785, Wave spectrum, Beijing, China) were used for the fluorescence excitation of ICG, and the infrared light from the sources was transmitted to the optical fibers for irradiation through four ports of the lighting mount. The image acquisition unit included an optical filter, focusing lens system, and image acquisition sensor. Further, a 785 nm notch filter was applied to prevent the reduction in the fluorescence signal-to-noise ratio owing to the specular reflection of the laser diodes. The focusing lens system employed a lens that transmitted visible light and fluorescent wavelength bands and was configured to obtain images through a common path for spatio-temporal synchronization of visible and NIR fluorescence images. In addition, the lens system was configured by setting the working distance at 30 cm from the front of the image head, ensuring that it did not interfere with the movement of the user in image-guided surgery and image diagnosis. For accurate focus adjustment of the acquired image, the imaging head is composed of a focus adjusting lens system. The focus of the image can be adjusted by rotating the focus adjustment ring located at the bottom of the imaging head for ease of user use. The image acquisition sensor comprised two sensors with high quantum efficiency in the band to simultaneously acquire visible and NIR images. Thereafter, the two images in the visible and NIR bands were separated via a dichroic prism with a cut-off wavelength of 700 nm inside a single camera and acquired by each of the two CMOS image sensors. CMOS image sensor, which converts light into electrical signals and images them, is suitable for dual sensor camera because CMOS image sensor has higher integration and less heat generation than the charged coupled device image sensor.

### 2.2. System Characterization

#### 2.2.1. Optical Characteristics

The optical properties of the proposed system were analyzed to evaluate its performance. The field-of-view (FOV), spatial resolution, and intensity of the infrared excitation light source were measured. The FOV was evaluated after acquiring images using a fixed focal length lens with a focal length of 8.5 mm and a ruler at a distance of 30 cm from the bottom of the imaging head. Further, to measure the spatial resolution, the USAF 1951 target (R3L3S1N, Thorlabs, Newton, NJ, USA) was placed at a distance of 30 cm from the imaging head to acquire the image, and the line profile of the captured image was analyzed. The aperture of the lens was fixed at the maximum opening and the f-number was 1.3. Finally, the flux rate of the NIR excitation light was measured by setting the currents of the two LDs to 1.5 A. A photodetector (S122C, Thorlabs, Newton, NJ, USA) with a 9.5 mm diameter of the detector area was placed in the center of FOV at a distance of 30 cm from the imaging head, and measurements were performed using a power meter (PM100D, Thorlabs, Newton, NJ, USA).

#### 2.2.2. Sensitivity and Signal-to-Background Ratio (SBR)

The sensitivity of the system to NIR signals was determined using the fluorescence signal according to the concentration of ICG (RFP0815, Bioacts, Incheon, Korea). The fluorescent signal increased when ICG was dissolved in polar solvents, such as dimethyl sulfoxide (DMOS), compared to nonpolar solvents and water [20]. DMSO was also identified as a highly stable and superior solvent for ICG [21,22]. Thus, ICG was diluted with ≥99.9% DMSO (Sigma–Aldrich, Darmstadt, Germany) and seven samples with concentrations ranging from 0.01 to 10 μM were used. Further, the current of the NIR LDs was set to 1.5 A, the voltage of the white LEDs was set to 12 V, and the experiment was performed with the room light turned on. Camera gain was fixed at 1.0, and images were obtained at exposure times of 10, 20, and 30 ms. Following repeated acquisition of images for each sample concentration, the grayscale average value of 5000 pixels in the sample area was calculated.

Thereafter, to optimize the acquisition of fluorescence images, the signal-to-background ratio according to the exposure time, gain, and sample concentration was investigated. The exposure time of the camera was adjusted from 5 to 40 ms, and gain was adjusted from 1 to 3; subsequently, fluorescent images were obtained for samples with ICG concentrations of 0.1, 0.5, 1, and 5 μM. An electric current of 2 A was applied to the NIR LDs. Further, a voltage of 12 V was applied to the white LEDs, with the room light turned on. Thereafter, background images were obtained from the white paper without ICG samples according to the exposure time and gain. In addition, to investigate the change in the background value due to ambient lighting, the image was acquired by turning the 12 W white LED and room lights on and off. Subsequently, the signal-to-background ratio was calculated by dividing the grayscale average value of 5000 pixels in the ICG-DMSO sample of the fluorescent image by the grayscale average value of all pixels of the background image.

(1) $$\mathrm{SBR}\;=\frac{\mathrm{average}\;\mathrm{of}\;5000\;\mathrm{pixels}\;\mathrm{in}\;\mathrm{the}\;\mathrm{sample}}{\;\mathrm{average}\;\mathrm{of}\;\mathrm{all}\;\mathrm{the}\;\mathrm{pixels}}\;$$

#### 2.2.3. Laser Diode Safety Test

The MTT assay method was used to evaluate the toxicity of light sources to cells [23]. One hundred μL of 4 × 10^3^ normal human dermal fibroblast cells (ATCC, Gaithersburg, MD, USA) was dispensed per well in a 96-well plate and cultured in an incubator maintained with 5% CO_2_ for 24 h. The plates were then irradiated for 10, 20, and 30 min, respectively, with a light source module wherein 2.3 mW/cm^2^ intensity 785 nm LD and white LEDs were arranged. Following incubation for 24 h, 50 μL of MTT [3-(4,5-dimethylthiazol-2-yl)-2,5-diphenyltetrazolium bromide] solution (2 mg/mL, Sigma-aldrich, St. Louis, MO, USA) dissolved in Dulbecco’s phosphate-buffered saline (DPBS) was added to each well and cultured in a 5% CO_2_ incubator maintained at 37 °C for 4 h. Thereafter, all the culture media was removed from each well, followed by the addition of 150 μL of DMSO and shaking for 30 s with a microplate mixer (Amersham, UK) to dissolve the formazan well. Subsequently, absorbance was measured at 540 nm using a microplate reader (Biochorom, Cambridge, UK). The survival rate was calculated using the following equation:

(2) $$\mathrm{Cell}\;\mathrm{Viability}\;\left(\%\right)=\frac{\mathrm{Mean}\;\mathrm{optical}\;\mathrm{density}\;\mathrm{in}\;\mathrm{test}\;\mathrm{well}}{\mathrm{Mean}\;\mathrm{optical}\;\mathrm{density}\;\mathrm{in}\;\mathrm{control}\;\mathrm{well}}\times 100$$

A biopsy was performed after irradiating the light source to the skin of the eyes and back of the rats to evaluate the toxicity of the light source to the eyes and skin. This animal study was conducted under the supervision of the Laboratory Animal Steering Committee of Dankook University, in accordance with an approved institutional protocol (approval number: DKU-20-041). For anesthesia, 6-week-old male Sprague-Dawley (SD) rats (weight: 185 g, Nara Biotech, Seoul, Korea) were administered isoflurane (Hana Pharm, Seoul, Korea), oxygen, and nitrogen through the nose, followed by irradiation with a light source placed 15 cm above the rat with an output of 2.4 mW/cm^2^ to the eyes and with hair removed for 30 min.

Owing to the characteristics of the eye and skin tissues, each tissue was prepared as a syngeneic section and a paraffin section, respectively. To prepare a section, the eyeball and skin tissue were extracted and stored in 10% (*v/v*) formaldehyde. In the case of the eyeball, to make a frozen section, pre-treatment was performed with saccharose (Duksan General Science, Seoul, Korea), and then the tissue was hardened using an optimal cutting temperature compound (Sakura Finetek, Torrance, CA, USA). Subsequently, the sections were cryosectioned to a thickness of 4 μm, whereas the skin tissue was embedded in paraffin and sectioned at a thickness of 4 μm using a microtome (RM2125, Leica, Germany) after pretreatment with ethanol. Each sectioned tissue sample was stained with hematoxylin and eosin for 1 min and 20 sec, respectively. Subsequently, images were obtained using a bright field microscope (BX53, Olympus, Tokyo, Japan) and compared with those of the control group.

### 2.3. In Vivo Imaging Test

ICG lymph node detection experiments were conducted at the Institute of Cancer Research Mice (ICR) to verify the in vivo performance of the fluorescence imaging system. This animal study was conducted under the supervision of the Laboratory Animal Steering Committee of Dankook University, in accordance with an approved institutional protocol (approval number: DKU-20-041). In this experiment, 80 μg of an anesthetic solution mixed with medetomidine hydrochloride in a 1:3 ratio and physiological saline diluted 1:9 was intramuscularly injected through the anterior part of the quadriceps muscle of 7-week-old male ICR mice weighing 20 g. Further, ICG at a concentration of 5 μg/mL was injected between the toes of the hind limbs. Five min after ICG injection, the skin was incised in the groin and the inguinal lymph nodes were confirmed via imaging. The camera gain was set to 3, with an exposure time of 5000 μs. 

Subsequently, the lymph nodes were excised and the fluorescence signal in the excised lymph nodes was confirmed. The video of the lymph node dissection experiment is presented in Supplementary Video S1.

## 3. Results

### 3.1. System Characteristics

#### 3.1.1. Optical Characteristics

**Figure 3:**
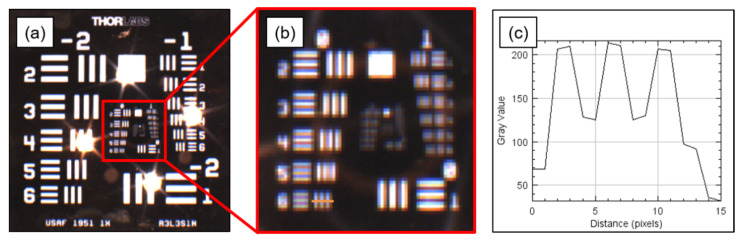
Spatial resolution test of the system. (**a**) Color image of USAF 1951 resolution target at 30 cm working distance. (**b**) Digitally magnified and cropped image of (**a**). (**c**) Line profile of element 6 on group 0 of the resolution target.

The image acquisition working distance of the developed system was 300 mm and the FOV was 206 mm × 154 mm. Further, the number of pixels in the camera was 1440 × 1080, and the resolution per pixel (μm/pixel) was 143 μm in the corresponding FOV. Figure 3 describes the spatial resolution test of the system. It was measured as 562 μm by analyzing the line profile of the resolution target image. When currents of 1.5 and 2.0 A were applied to two infrared laser diodes, the intensity of the light source in the surgical plane 30 cm from the end of the fiber was measured at 500 ± 10 μW/cm^2^ and 700 ± 15 μW/cm^2^, respectively.

#### 3.1.2. Sensitivity

**Figure 4:**
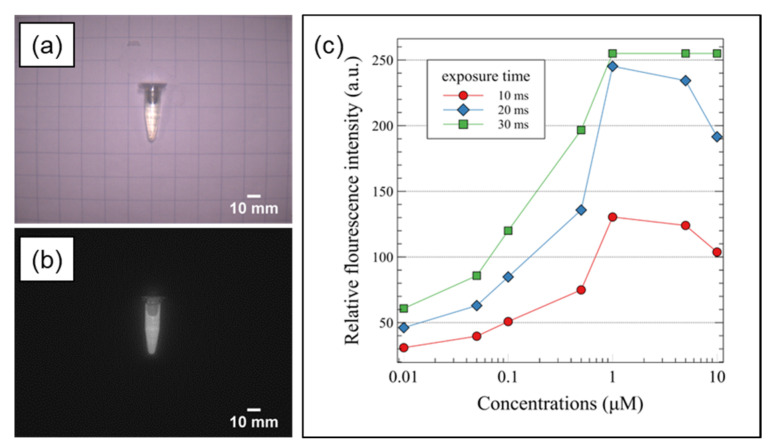
Sensitivity evaluation of the system (**a**) Color image of ICG solution (**b**) NIR image of ICG solution (**c**) Average fluorescence intensity from various concentrations of ICG and exposure times.

Figure 4 shows the sensitivity of this system to the fluorescence signal according to the ICG-DMSO sample concentration. Figure 4a,b show the VIS and NIR images of the sample, respectively. Figure 4c shows the grayscale average value of 5000 pixels in the sample area according to the sample concentration in the NIR image. With an increase in the concentration, the fluorescence signal increased, and the strongest signal was observed for the 1 μM sample. Furthermore, with an increase in the exposure time, the fluorescence signal increased, and the signals of the 1, 5, and 10 μM samples were saturated at an exposure time of 30 ms.

#### 3.1.3. Signal-to-background Ratio (SBR)

**Figure 5:**
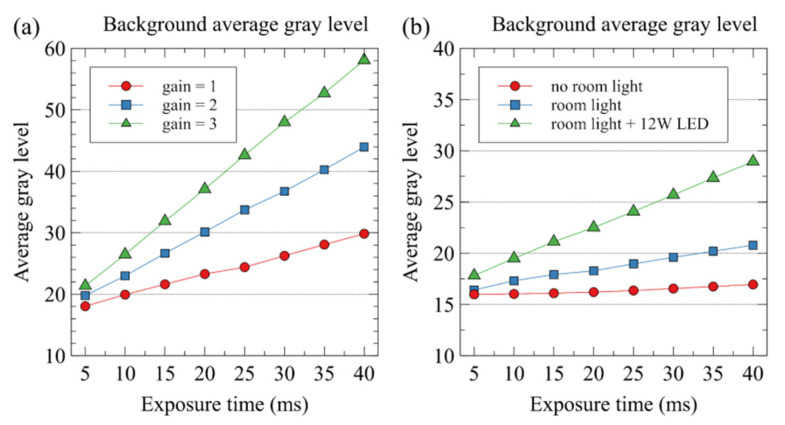
Background average gray level in different gain (**a**) and lighting conditions (**b**). (**a**) Background noise measured with 12 W LED lights and room lights on. (**b**) Background image measured with gain of 1.

Figure 5 shows the average gray level of all the pixels of the NIR background image. As expected, with the increase in the gain and exposure time, the average grey level of the background image increased. Moreover, with the addition of lighting, the background gray-level value also increased.

**Figure 6:**
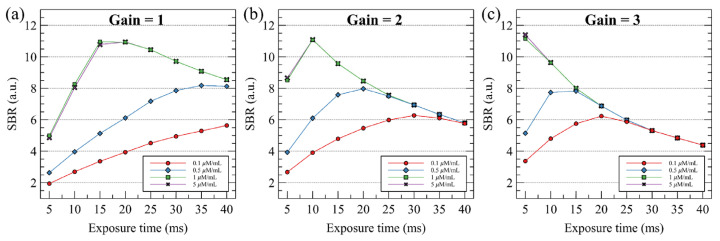
Signal-to-background ratio (SBR) measurements. SBR are measured in different of gain, exposure time, and concentrations of ICG solution. Each line in the caption describes the concentration of the ICG solution.

Figure 6 shows the signal-to-background ratio according to the sample concentration and exposure time for the gain values of 1, 2, and 3. In high-concentration samples (1 and 5 μM), the optimal SBR values were observed at exposure times of—15–20, 10, and 5 ms exposure times as the gain values were changed to 1, 2, and 3, respectively. The maximum SBR was observed at the start of fluorescence signal saturation as the gain and exposure time increased. Subsequently, when the exposure time or gain was increased, the background image noise increased and the SBR gradually decreased. In the low-concentration sample (0.1 μM), the optimum SBR value could not be obtained at a gain of 1. However, when the gain was set to 2 and 3, the optimum SBR value was obtained at exposure times of 30 and 20 ms, respectively.

#### 3.1.4. Laser Diode Safety Test

**Figure 7:**
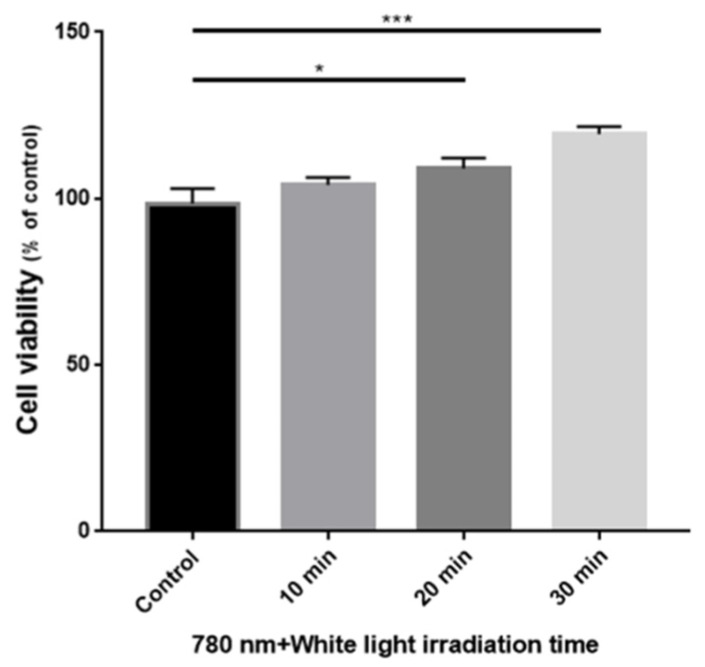
Cell viability by MTT assay by 785 nm LD and white LED irradiation time. *, and *** indicates significant differences (*p* < 0.05, *p* < 0.001, respectively).

Figure 7 shows the results of the MTT assay performed to evaluate the toxicity of the light source. Compared to the control sample, the cell viability of the samples irradiated with a light source for 10, 20, and 30 min was high, indicating that no cell necrosis due to photostimulation occurred.

**Figure 8:**
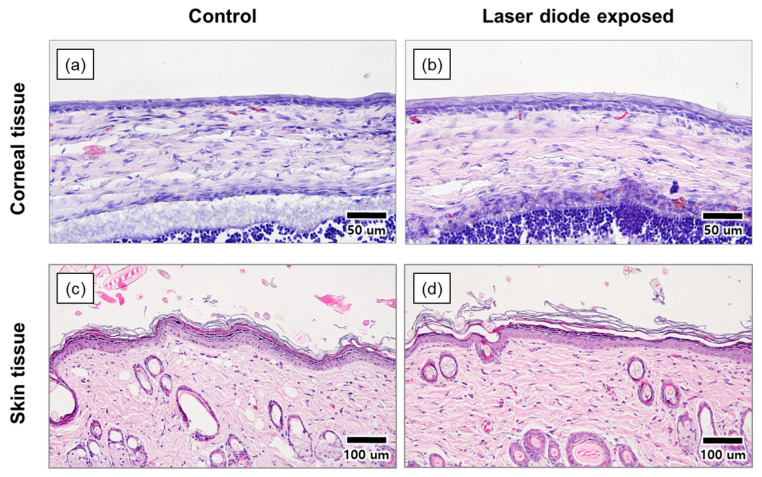
(**a**,**b**) Corneal, (**c**,**d**) skin tissue images of the Sprague Dawley rat. (**b**,**d**) tissue images are obtained after 30 min of irradiation of NIR LDs in the rat to the eyes and back skin.

Figure 8 shows images of the back skin and eyes of SD rats observed under a microscope after staining the cell tissues irradiated with 785 nm LD at an intensity of 2.4 mW/cm^2^ for 30 min. In the eyeball images, no damage to the corneal epithelium was found compared to the control group. Further, considering the skin-tissue images, the boundaries of each layer and hair follicle were histologically normal. Thus, these experiments showed no biological toxicity owing to the 785 nm LD used as a fluorescence excitation light source.

### 3.2. In Vivo Imaging

**Figure 9:**
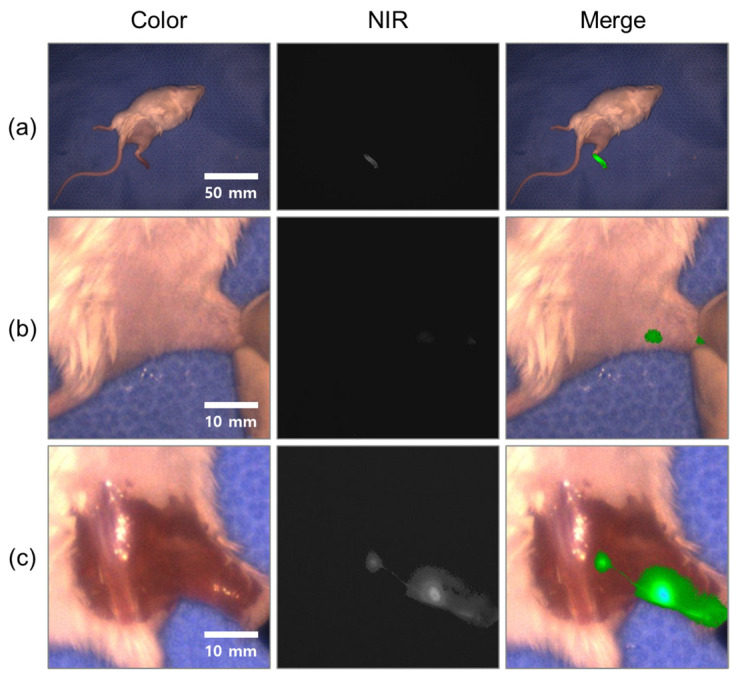
In vivo evaluation of the system. (**a**) ICR mouse 5 min after injection of ICG (**b**) right groin of the mouse (**c**) iliac lymph node and popliteal lymph node of the mouse after skin incision.

Figure 9 shows an image of the in vivo performance of the system. ICG was injected between the toes of the mouse, with the fluorescence signal in the right groin confirmed 5 min later. Subsequently, the skin was incised to obtain detailed images of the lymph nodes. The camera gain was 3, the exposure time was 5000 μs, and the system captured images at 30 frames per second (fps). Figure 9b,c show digital fivefold magnification and cropped images showing the iliac lymph node and popliteal lymph node of the mouse before and after skin incision. The fluorescence signal in the lymphatic tube was photographed in the image obtained after skin incision. Further, the SBR of the popliteal lymph node was measured to be 1.8 before skin incision and 5.6 after incision. Thus, this showed that the developed NIR fluorescence imaging system was suitable for ICG-based image-guided surgeries.

## 4. Discussion

The clinical application of NIR fluorescence imaging systems is expanding based on real-time imaging of biological tissues using fluorescence contrast agents. Furthermore, the development of new types of fluorophores for specific cancer diagnosis and treatment has increased the potential of these imaging systems [24,25,26].

When developing an NIR fluorescent imaging system, color-fluorescent image matching for user convenience, high frame images without blurring according to motion, working distance, device miniaturization that does not interfere with surgery, and an appropriate field of view are required. In addition, optical, electrical, and mechanical safety certifications for the development of medical imaging devices are required for certification for clinical application.

In this study, a compact NIR fluorescence imaging system using a dual-CMOS camera was proposed. This system provides color, NIR fluorescence, and overlay images of two images simultaneously for user convenience. In addition, for image analysis, the fluorescent image display method can be changed to a boundary mode and a color-map mode, and threshold of the fluorescent signal can be adjusted to allow users to accurately determine the location and size of the target. Further, it provides smooth images at a frame rate of 30 fps, while detecting fluorescence signals for ICG as low as 10 nM. Moreover, the in vivo test on ICR mice showed the possibility of clinical application such as sentinel lymph node detection of various cancers such as breast, gastric, rectal, anal, colon, colorectal, bladder, cervical, vulvar cancer, and parathyroid detection in thyroidectomy, and visualization of skin perfusion [8,9,10,11,12,13,14,27,28,29].

Factors that affect the SBR ratio in this system include the intensity of the excitation light source, concentration of the sample, exposure time and gain of the camera, and surrounding light source. In the case of a high-concentration sample, the optimal fluorescence image can be obtained only by adjusting the exposure time, whereas, in the low-concentration sample, the optimal image can be obtained by adjusting the exposure time and gain value. In clinical trials, if the target sample is deep, the attenuation of the fluorescence signal increases. In this case, the optimal image can be obtained by adjusting the exposure time from 30 to 40 ms and subsequently increasing the gain step by step.

Based on the image analysis of the USAF 1951 resolution target, the spatial resolution of the system was approximately 560 μm. However, a higher resolution may be required, depending on the application. To this end, there is a method to replace a camera with a higher number of pixels, and there is a commercial product with four times more pixels in the same structure as the camera we used. Otherwise, a lens with a longer focal length can be used; however, there is a tradeoff with the FOV.

However, there are image quality issues in color and fluorescence images of the developed system. The color images appeared rather dark due to insufficient white light and room light for high SBR fluorescent images. By imaging the difference between on and off images of the infrared light source after synchronizing the infrared light source with the infrared camera, color images of appropriate brightness can be obtained while keeping the high SBR of fluorescence images. In Figure 9c, the bright part of the fluorescent image looks blurred, but we can also see thin lymphatic vessels without blurring. In Figure 9a, there is no blur even though the brightness of the fluorescent signal of the mouse foot is bright. Blurring occurs because infrared light is scattered when it comes out of the target sample, so blurring occurs more deeply in the target sample, which is also the limitation of near-infrared fluorescence imaging devices. For accurate resection surgery, a procedure to check the boundary of the target by adjusting the threshold of the fluorescence signal is required by the surgeon.

Since the light source is irradiated with a Gaussian distribution, the intensity of the fluorescent signal depends on the position of the sample. To minimize this effect, the angle of the four infrared ports were adjusted for uniform irradiation. However, there is insufficient infrared light near the edges. To compensate with amplifying the fluorescent signal, there are functions to set the gain of the camera and set the threshold. For the clinical version of the system, for those unfamiliar with these systems, we plan to mark the boundaries where the light will be sufficiently irradiated.

It is difficult for users unfamiliar with these devices to adjust the gain and exposure time of the camera to obtain images with the optimal SBR. An auto-exposure control algorithm is applied to the cell phones and digital cameras that people use in their daily lives to easily obtain good-quality pictures. However, because most of the infrared images of the fluorescence imaging system are dark, except for the part where the fluorescence signal is generated, the application of the general auto-exposure control algorithm is challenging. Accordingly, investigations for an auto-exposure control algorithm dedicated to fluorescence imaging systems will be conducted in the future.

## 5. Conclusions

This study proposed a fluorescence imaging system that uses a single dual-CMOS-sensor camera. Using two separate cameras renders a system bulky and requires considerable effort to match the images of the two cameras; however, this study designed a compact system using a single dual-CMOS camera. Further, to evaluate the performance of the system, its optical performance was measured, and the sensitivity and signal-to-background ratio for a fluorescent signal according to the concentration of the ICG sample and the exposure time and gain of the camera were measured. Moreover, through light source toxicity tests on fibroblast cells and the eyes and skin of SD rats, the laser safety of the system was demonstrated. Furthermore, the clinical applicability of the system was demonstrated through a detection and extraction test of the groin lymph nodes of ICR mice.

## Data Availability

Not applicable.

## Supplementary Materials

The following supporting information can be downloaded at: https://www.mdpi.com/article/10.3390/s22155597/s1, Video S1, lymph node dissection experiment.
